# The Efficacy and Safety of Acupuncture for the Treatment of Children with Autism Spectrum Disorder: A Systematic Review and Meta-Analysis

**DOI:** 10.1155/2018/1057539

**Published:** 2018-01-11

**Authors:** Boram Lee, Jihong Lee, Jin-Hong Cheon, Hyun-Kyung Sung, Seung-Hun Cho, Gyu Tae Chang

**Affiliations:** ^1^Department of Clinical Korean Medicine, Graduate School, Kyung Hee University, Kyung Hee Dae-ro 26, Dongdaemun-gu, Seoul 02447, Republic of Korea; ^2^Department of Korean Pediatrics, Hospital of Korean Medicine, Pusan National University, Geumo-ro 20, Mulgeum-eup, Yangsan-si, Gyeongsangnam-do 50612, Republic of Korea; ^3^Department of Pediatrics, College of Korean Medicine, Hospital of Semyung University, St. Sangbang 4-63, Chungju City, Chungcheongbuk-do 27429, Republic of Korea; ^4^Department of Neuropsychiatry, College of Korean Medicine, Kyung Hee University, Kyung Hee Dae-ro 26, Dongdaemun-gu, Seoul 02447, Republic of Korea

## Abstract

*Objectives. *We aimed to summarize and critically evaluate the available evidence regarding the efficacy and safety of acupuncture for children with autism spectrum disorder (ASD).* Methods.* We searched 13 databases for studies published up to December 2016. Randomized controlled trials (RCTs) evaluating the efficacy of acupuncture for children with ASD were included. Outcome measures were the overall scores on scales evaluating the core symptoms of ASD and the scores for each symptom, such as social communication ability and skills, stereotypies, language ability, and cognitive function. Effect sizes were presented as mean differences (MD).* Results.* Twenty-seven RCTs with 1736 participants were included. Acupuncture complementary to behavioral and educational intervention significantly decreased the overall scores on the Childhood Autism Rating Scale (CARS) (MD −8.10, 95% CI −12.80 to −3.40) and the Autism Behavior Checklist (MD −8.92, 95% CI −11.29 to −6.54); however, it was unclear which of the ASD symptoms improved. Acupuncture as a monotherapy also reduced the overall CARS score. The reported adverse events were acceptable.* Conclusions.* This review suggests that acupuncture may be effective and safe for pediatric ASD. However, it is not conclusive due to the heterogeneity of the acupuncture treatment methods used in the studies.

## 1. Introduction

Autism spectrum disorder (ASD) is a neurodevelopmental disorder with an etiology that remains incompletely understood. The core symptoms of ASD include persistent deficits in social communication and interaction and restricted, repetitive patterns of behavior, interests, or activities [1]. The prevalence of ASD as reported in various studies ranges from as low as 1 in 500 to as high as 1 in 50 [2, 3].

Many types of treatments are available for ASD, but none have yet been developed that effectively treat the core symptoms. Conventional treatments for ASD include pharmacological therapy and behavioral and educational interventions (BEI). However, risperidone, a commonly used medication for the treatment of maladaptive behaviors in ASD, has adverse effects such as weight gain, fatigue, drowsiness, and tremors [4, 5]. Furthermore, the majorities of high quality BEI require 20 to 40 hours of treatment per week, and take a long time to show benefits [6–8].

The National Center for Complementary and Integrative Health describe “complementary” as a therapy used alongside conventional medicine, and “alternative” as a therapy used in place of conventional medicine [9]. Recent studies have reported that approximately 88% of a large, geographically diverse sample of children with ASD had used complementary and alternative medicine (CAM) such as gluten-free casein-free diets, melatonin, or music therapy in either the past or recently [10]. Families of children with ASD may choose CAM to treat a variety of symptoms, such as hyperactivity, inattention, gastrointestinal symptoms, or sleep disturbances [11], or due to concerns about the adverse effects of conventional treatments [12].

Acupuncture is one of the most popular forms of CAM [13]. As a form of Traditional Chinese Medicine (TCM), acupuncture is also widely used in Western countries [14]. In TCM, the pathogenesis of ASD is theorized to result from derangement or insufficiency of the brain and mind, and dysregulation of the heart, liver, spleen, and kidney after birth [15]. Acupuncture is used to correct the disharmony of organ systems and is theorized to address the symptoms of ASD by stimulating acupoints that are related to organs or viscera. The physiological mechanisms by which acupuncture works for ASD seem to be very complicated and remain unclear. The possible mechanisms by which acupuncture affects ASD include regulation of neurotransmitters [16, 17], which have been shown to be disturbed in many people with ASD [18], modulation of expression and activation of brain-derived neurotrophic factor (BDNF), which is involved in the pathophysiology of ASD [19], and so on.

Prior to 2011, systematic reviews [20, 21] concluded that there was no evidence for the use of acupuncture in ASD, because of the low total number and low methodological quality of the studies. Because of this lack of evidence, acupuncture treatment could not be recommended in existing clinical guidelines for the treatment of ASD. Since then, however, many studies of acupuncture for pediatric cases of ASD have been published. The objective of this study was to summarize and critically evaluate the updated evidence for the efficacy and safety of acupuncture for the treatment of ASD in pediatric patients.

## 2. Methods

This systematic review and meta-analysis was conducted according to the guidelines in the Cochrane Handbook for Systematic Reviews of Interventions [22]. Reporting was conducted in accordance with the Preferred Reporting Items for Systematic Reviews and Meta-Analyses (PRISMA) guidelines [23]. The protocol of this review was registered in the International Prospective Register of Systematic Reviews, PROSPERO (CRD42017054544).

### 2.1. Search Strategy

Two authors (B. L. and J. L.) performed a comprehensive search of the following 13 electronic databases from their inception dates to 31 December 2016: six English databases (Cochrane Library, MEDLINE via EBSCO, EMBASE via Elsevier, AMED via EBSCO, CINAHL via EBSCO, and PsycARTICLES via ProQuest), two Chinese databases (Chinese National Knowledge Infrastructure (CNKI) and Wanfang Data), two Japanese databases (Japanese Institutional Repositories Online (JAIRO) and Citation Information by NII (CiNii)), and three Korean databases (Oriental Medicine Advanced Searching Integrated System (OASIS), Korean Traditional Knowledge Portal (KTKP), and KoreaMed). We also searched the reference lists of the relevant papers to identify additional trials. There was no restriction on language. In addition to the studies published in the journal, we also included grey literature, such as conference proceedings and degree theses.

The following search terms were used in MEDLINE: (autis^*∗*^ OR pervasive developmental disorder^*∗*^ OR childhood disintegrative disorder OR asperger^*∗*^ OR autism spectrum disorder OR child development disorders, pervasive) AND (acup^*∗*^ OR needl^*∗*^ OR trigger point OR body acupuncture OR scalp acupuncture OR tongue acupuncture OR auricular acupuncture OR pharmacopuncture OR bee OR acupuncture OR acupuncture therapy OR acupuncture points OR acupuncture, ear OR electroacupuncture). The details of search strategies used in all databases are presented in the Supplementary Material (Supplement 1).

### 2.2. Inclusion and Exclusion Criteria

#### 2.2.1. Types of Studies

We included randomized controlled trials (RCTs) that evaluated the efficacy of acupuncture for children with ASD, including those using a quasi-random method such as alternate allocation or allocation by birth date. We included both parallel and crossover studies.

#### 2.2.2. Types of Participants

We included patients with ASD under the age of 18 years, regardless of gender or race, diagnosed by standard criteria such as the Diagnostic and Statistical Manual of Mental Disorders (DSM) or the International Classification of Diseases (ICD). We accepted diagnoses by assessment tools such as the Autism Diagnostic Observation Scale (ADOS), Autism Diagnostic Interview-Revised (ADI-R), Childhood Autism Rating Scale (CARS), Chinese Classification of Mental Disorder (CCMD), and other validated tools. Studies on ASD were included even if they did not refer to the diagnostic criteria.

#### 2.2.3. Types of Interventions

We included studies on acupuncture involving the insertion of needles into traditional acupoints or into nonmeridian points as experimental interventions, regardless of the acupuncture treatment method. Studies that did not involve skin penetration, such as those using acupressure, were excluded. Control interventions included pharmacological interventions, BEI, and CAM, including combinations of two or more therapy types. We also included sham acupuncture, which refers to a needle placed in an area close to but not into acupoints, as a control intervention. Studies in which the other treatments were applied to both experimental and control groups in the same manner were also included. We excluded trials that compared only different forms of acupuncture.

#### 2.2.4. Types of Outcome Measures

The outcome measures were as follows: (1) the overall score on scales evaluating the core features of ASD, including CARS, the Autism Behavior Checklist (ABC^1^), the Aberrant Behavior Checklist (ABC^2^), the Autism Treatment Evaluation Checklist (ATEC), and the Ritvo-Freeman Real Life Rating Scale (RFRLRS); (2) social interaction skills, communication ability, or stereotypy, which are essential symptoms of ASD; and (3) language ability or cognitive function, which are associated features of ASD.

### 2.3. Study Selection and Data Extraction

After eliminating duplicate studies, two authors (B. L. and J. H. C.) independently screened the titles and abstracts of the searched studies to identify those that potentially met the inclusion criteria. Thereafter, the texts of the remaining articles were obtained and screened for eligibility by two authors (J. H. C. and H. K. S.).

One author (B. L.) extracted the data from the selected studies, and a different author (S. H. C.) reviewed the data. Discrepancies were resolved through discussion with a third author (G. T. C.). The extracted information included study design, country, demographic characteristics of the participants, diagnostic criteria, details of the experimental and control interventions, outcomes, adverse events, and information for assessing the risk of bias. We contacted the primary authors of the included studies via email if additional information was needed.

### 2.4. Quality Assessment

Two authors (J. H. C. and H. K. S.) independently evaluated the methodological quality of the included studies, using the Cochrane Collaboration's tool [22]. The following characteristics were assessed: random sequence generation, allocation concealment, blinding of participants and personnel, blinding of outcome assessments, incomplete outcome data, selective reporting, and other bias. Each domain was evaluated and categorized into three groups: “low risk,” “unclear,” or “high risk.” If the relevant information was not mentioned in the article, we evaluated the domain as “unclear.” Disagreements between the review authors over the risk of bias were resolved by discussion, with the participation of another author (J. L.).

### 2.5. Statistical Analysis

For studies using the same type of intervention, comparator, and outcome measure, quantitative synthesis was conducted by meta-analysis using the Review Manager software, version 5.3 (Cochrane, London, UK). Descriptive analysis was conducted when the number of reported studies was only one, or when it was considered that heterogeneity was too high for the results to be synthesized. For the meta-analyses, risk ratios (RR) and 95% CIs were calculated for dichotomous data, and mean differences (MD) and 95% CIs were used for continuous data. We used pre-post differences or end-point scores as outcome measures, presented for each included study. If among the included studies there were some measuring the change in a value, and others measuring the final value, of the same outcome measures, we planned to synthesize the data by calculating the final value as the initial value plus the change, if possible. We examined heterogeneity among the studies using the Higgins *I*^2^ test. We considered *I*^2^ ≥ 50% to be indicative of substantial heterogeneity, and *I*^2^ ≥ 75% to be indicative of serious heterogeneity. In the meta-analyses, a random effects model was used when the heterogeneity was significant, while a fixed effects model was used when the heterogeneity was not significant or the number of studies included in a meta-analysis was very small, in which case estimates of interstudy variance have poor accuracy [24].

## 3. Results

### 3.1. Study Description

#### 3.1.1. Literature Search

**Figure 1:**
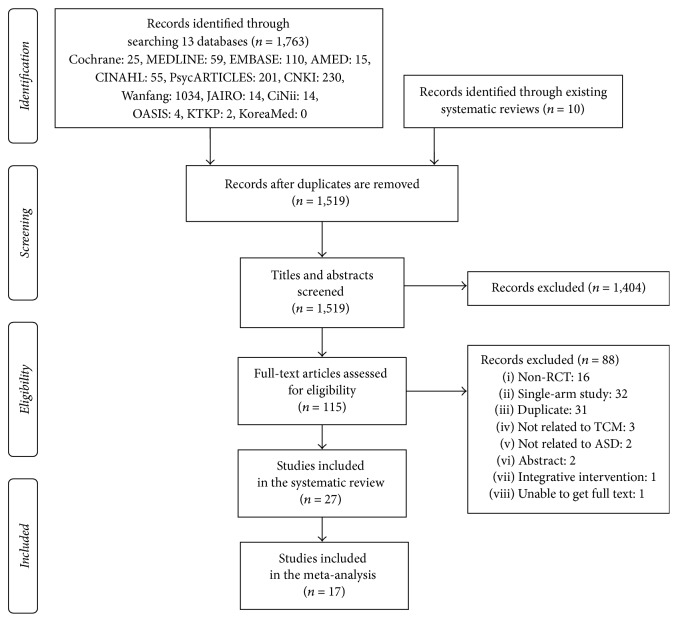
PRISMA flow chart of the literature screening and selection process.

We identified 1763 records through a database search, and 10 additional records from the reference lists of relevant papers. After removing duplicates, 1519 records remained. A review of the titles and abstracts excluded 1404 records. After assessing the full text of the remaining 115 articles, we finally included 27 articles [25–51] in the systematic review and 17 articles [27, 29, 32–36, 38–43, 45–47, 49] in the meta-analysis (Figure 1).

#### 3.1.2. Study Characteristics

Table 1 describes the characteristics of the included studies. The most common diagnostic reference was the DSM. Five studies [25, 40–43] received institutional review board approval before the study was conducted. Consent forms were obtained from research participants in 22 studies [25–30, 32, 34–43, 46–49, 51]. Only 4 studies [40–43] registered the protocol before the trial.

Experimental interventions involved manual acupuncture in 19 trials [25–28, 31–34, 36, 39, 42–48, 50, 51], electroacupuncture in 6 trials [30, 35, 37, 38, 41, 49], and both manual acupuncture and electroacupuncture in 2 trials [29, 40]. The most frequently used acupoints were GV20 [25, 26, 37, 38, 40, 49] and EX-HN3 [26, 29, 37, 38, 40, 41], used in six trials each. The depth of insertion ranged from 0.3 cm to 1.5 cun, and the heterogeneity was high, depending on the location of the acupoints. (Cun, a traditional Chinese unit of length, corresponds to 3.0303030 cm [52].) The most commonly used needle retention time was 30 minutes; the most frequent number of treatment sessions was 120; the most commonly used frequency and duration of acupuncture treatment were 5 times/week and 8 weeks. There was no follow-up study after treatment in any of the studies. Details of the acupuncture treatment methods used can be found in Table 2.

As outcome measures, CARS was evaluated in 11 trials [27, 29, 30, 32–36, 39, 45, 46], ABC^1^ was evaluated in 5 trials [30, 32, 34, 38, 49], and ABC^2^ [29, 40, 41], ATEC [29, 40, 43], and RFRLRS [40–42] were each evaluated in 3 trials. Social interaction skills were assessed in 9 studies [29, 38, 40–43, 46, 48, 49], communication skills in 6 [29, 40, 41, 43, 46, 48], stereotypy in 2 [40, 41], language skills in 13 [25, 27, 28, 30, 38–43, 47–49], and cognitive function in 9 [28, 29, 31, 40–43, 47, 48], using a variety of outcome measures.

### 3.2. Quality Assessment

**Figure 2:**
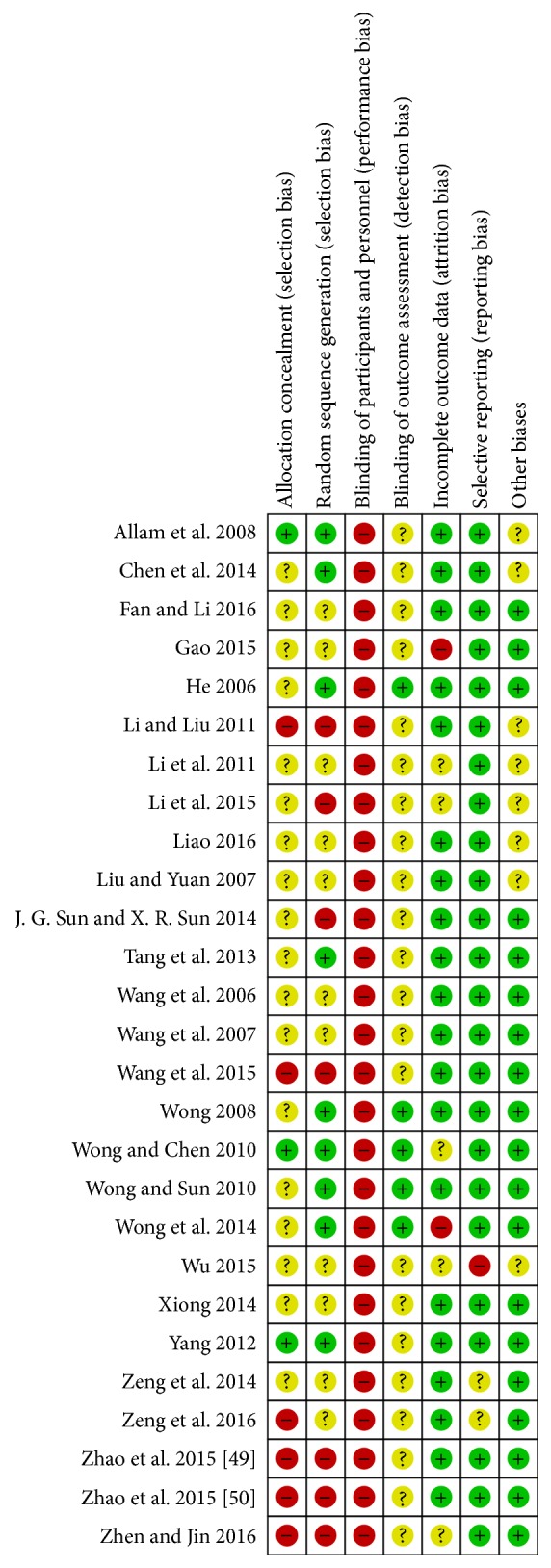
Summary of risk of bias in all included studies; “+” low risk; “?” unclear risk; “−” high risk.

Of the 27 trials included, only 3 [25, 41, 46] used sealed or opaque envelopes and were rated as having “low risk” of bias in allocation concealment. A further 6 studies [30, 39, 48–51] were rated as “high risk” because they did not use appropriate concealment methods, and the remaining 18 studies [26–29, 31–38, 40, 42–45, 47] were evaluated as “unclear” due to lack of information. Nine studies [25, 26, 29, 36, 40–43, 46] used adequate methods of random sequence generation, using a randomization table or computer-generated randomization number. Because it is difficult to blind participants and therapists to acupuncture manipulation procedures, all studies were evaluated as “high risk” in the performance bias category. Only five trials [29, 40–43] reported the blinding of outcome assessment, and the remaining studies [25–28, 30–39, 44–51] were evaluated as “unclear” due to lack of information. Twenty studies [25–27, 29, 30, 33–40, 42, 45–50] had “low risk” of attrition bias, but five [31, 32, 41, 44, 51] were “unclear” because they did not provide statistical methods to deal with missing values. In two studies [28, 43], there were also dropouts, but they were rated “high risk” because of statistical methods using per-protocol analysis. Twenty-four studies [25–43, 45, 46, 49–51] were assessed as “low risk” in reporting bias, while two [47, 48] reported only effective rates without presenting raw data, and one [44] did not report all scheduled results. We contacted the primary authors of the studies [47, 48] to obtain the raw data via email, but did not receive the data. Except for the eight studies [25, 26, 30–34, 44] with possible baseline imbalances among participants in the experimental and control groups, the remaining studies [27–29, 35–43, 45–51] were evaluated as “low risk” in the other bias domain. Methodological assessments for each included study are presented in Figure 2.

### 3.3. Efficacy of Acupuncture as a Complementary Therapy

#### 3.3.1. Complementary Therapy to BEI

Sixteen studies [25, 26, 29, 31–33, 35–38, 40, 43, 45, 47, 49, 51] compared acupuncture plus BEI to BEI alone.

**Figure 3:**
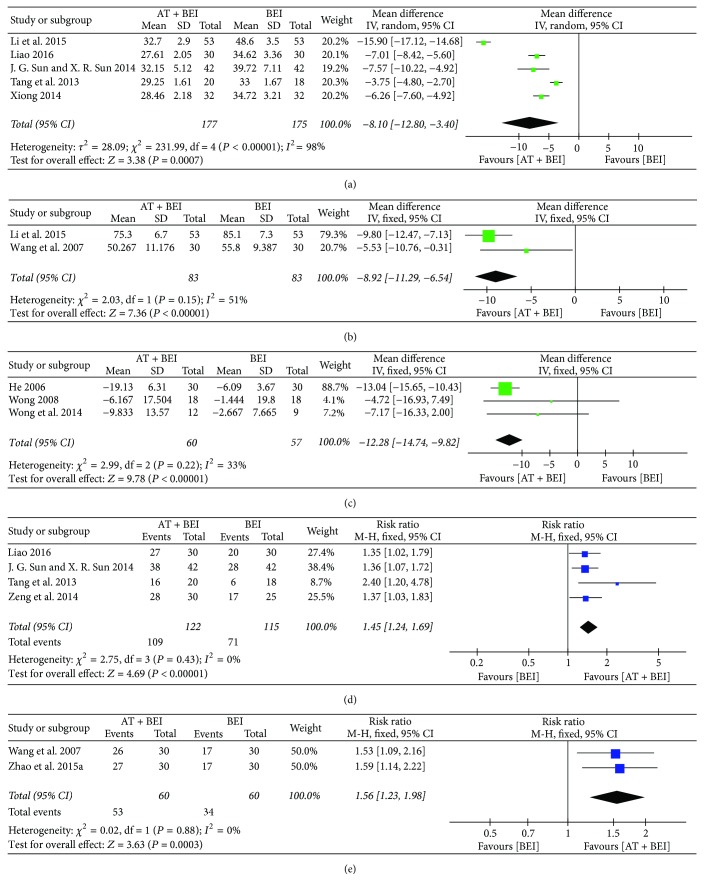
*Forest plots for comparison of acupuncture plus BEI versus BEI alone. (a) Overall CARS score. (b) Overall ABC* ^*1*^ *score. (c) Overall ATEC score. (d) TER based on CARS score. (e) TER based on ABC score.* ABC^1^, Autism Behavior Checklist; AT, acupuncture treatment; ATEC, Autism Treatment Evaluation Checklist; BEI, behavioral and educational interventions; CARS, Childhood Autism Rating Scale; TER, total effective rate.

*(1) Overall Scores on Scales Evaluating the Core Features of ASD.* When acupuncture was added to BEI, the meta-analyses showed that reductions of the overall CARS [32, 33, 35, 36, 45], ABC^1^ [32, 38], and ATEC scores [29, 40, 43] were higher after treatment than for BEI alone (CARS, MD −8.10, 95% CI −12.80 to −3.40, *I*^2^ = 98%; ABC^1^, MD −8.92, 95% CI −11.29 to −6.54, *I*^2^ = 51%; ATEC, MD −12.28, 95% CI −14.74 to −9.82, *I*^2^ = 33%). Meta-analyses showed higher values in the acupuncture plus BEI groups than in the control groups in the studies that evaluated improvements in CARS [33, 35, 36, 47] and ABC^1^ [38, 49] scores using the total effective rate (TER) (CARS-TER, RR 1.45, 95% CI 1.24 to 1.69, *I*^2^ = 0%; ABC^1^-TER, RR 1.56, 95% CI 1.23 to 1.98, *I*^2^ = 0%) (Figure 3).

One study [29] found that the total ABC^2^ scores were improved in the acupuncture plus BEI group compared with the BEI group (*P* < 0.01), but in another study [40] the differences were not significant (*P* = 0.205). One study [29] did not present the standard deviation, thus quantitative synthesis could not be performed. In one study [40], which evaluated the overall RFRLRS score, no significant difference was found between the two groups (*P* = 0.752). 

**Figure 4:**
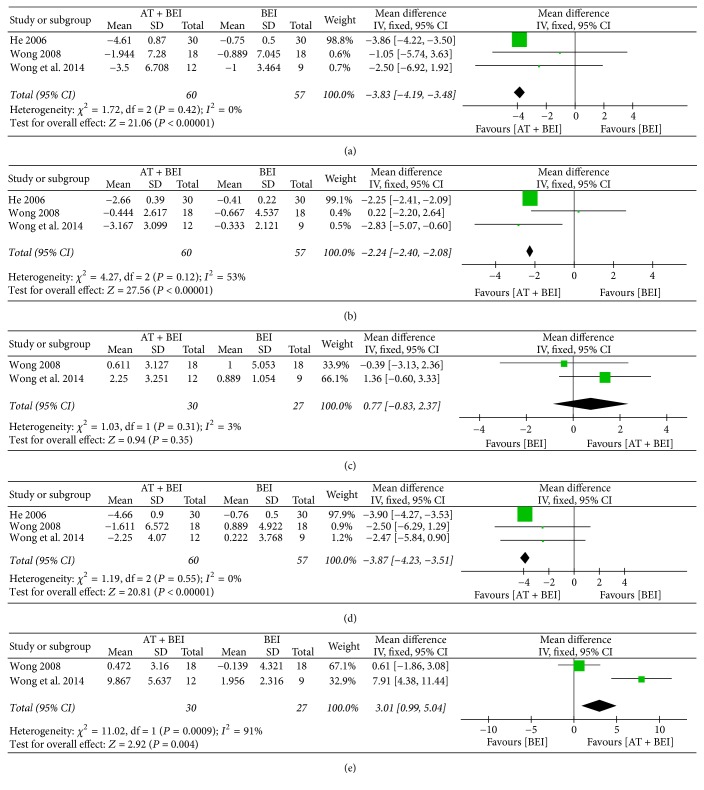
*Forest plots for comparison of acupuncture plus BEI versus BEI alone. (a) Social interaction skill (ATEC). (b) Communication ability (ATEC). (c) Language ability (SPT). (d) Cognitive function (ATEC). (e) Cognitive function (WeeFIM).* AT, acupuncture treatment; ATEC, Autism Treatment Evaluation Checklist; BEI, behavioral and educational interventions; SPT, Symbolic Play Test; WeeFIM, Functional Independence Measure for Children.

*(2) Social Interaction Skills.* Five studies [29, 38, 40, 43, 49] assessed social interaction skills, among which three [29, 40, 43] used a subscale of the ATEC and showed improvements when acupuncture was additionally used (MD −3.83, 95% CI −4.19 to −3.48, *I*^2^ = 0%) (Figure 4).

Of the two studies [38, 49] that assessed social interaction skills using ABC^1^, Zhao et al. [49] found significant improvement in the acupuncture group compared with the controls (*P* = 0.019), while no such effect was found by Wang et al. [38] (*P* > 0.05). We could not do a meta-analysis because of considerable baseline variations between the studies. Wong [40] reported that there were no significant differences between the two groups when social interaction was assessed by a subscale of the ADOS (*P* = 0.987) or RFRLRS (*P* = 0.268). 

*(3) Communication Ability.* Three studies [29, 40, 43] evaluated communication skills assessed with a subscale of the ATEC and reported improvements in the acupuncture plus BEI groups compared with the BEI groups (MD −2.24, 95% CI −2.40 to −2.08, *I*^2^ = 53%) (Figure 4).

There was no difference between the two groups in one study [40], in which subscales of the ABC^2^ and ADOS were used to assess communication skills (*P* = 0.183, *P* = 0.604; resp.).

*(4) Stereotypy.* There was no difference between the two groups in one study [40] in which a subscale of the ABC^2^ was used to assess stereotypy (*P* = 0.484).

*(5) Language Ability.* Six studies [25, 38, 40, 43, 47, 49] assessed language ability. Two studies [40, 43] evaluated it using the Symbolic Play Test (SPT), and found no significant difference (MD 0.77, 95% CI −0.83 to 2.37, *I*^2^ = 3%) (Figure 4).

Two studies [38, 49] evaluated language ability using a subscale of the ABC^1^, one of which [49] showed a significant improvement in the acupuncture plus BEI group compared with the controls (*P* < 0.05), while the other [38] did not (*P* > 0.05). We could not conduct a meta-analysis due to the heterogeneity of the baseline values. Zeng et al. [47] found a significant improvement in language ability in the acupuncture group compared with the controls, when assessed with TER calculated using the Psychoeducational Profile (PEP) (*P* < 0.05), while no significant difference was found in other studies evaluated with the Arabic language test [25], Peabody Picture Vocabulary Test [38], Reynell Developmental Language Scales (RDLS) [43], and RFRLRS [40]. 

*(6) Cognitive Function.* Five studies [29, 31, 40, 43, 47] measured cognitive function, among which three [29, 40, 43] used a subscale of the ATEC and showed significant improvements in the acupuncture plus BEI group compared with the BEI group (MD −3.87, 95% CI −4.23 to −3.51, *I*^2^ = 0%). The pooled results assessing cognitive function with the Functional Independence Measure for Children (WeeFIM) [40, 43] showed a comparative benefit of using acupuncture in conjunction with BEI (MD 3.01, 95% CI 0.99 to 5.04, *I*^2^ = 91%) (Figure 4).

Two studies [31, 47] reported greater improvements in cognitive function, evaluated by the PEP-revised and TER of the PEP, respectively, in the acupuncture plus BEI group than in the control group (*P* = 0.05, *P* = 0.02; resp.).

#### 3.3.2. Complementary Therapy to Pharmacotherapy

One study [50] compared acupuncture plus pharmacotherapy to pharmacotherapy alone, and descriptive analysis was performed. The study showed that the addition of acupuncture to risperidone improved abnormal behaviors, including stereotypy assessed by the TER, compared with risperidone alone (*P* < 0.05).

#### 3.3.3. Complementary Therapy to BEI Combined with Music Therapy

Two studies [30, 48] compared acupuncture plus a combination therapy including both BEI and music therapy, which is a form of CAM, to the combination therapy. Only descriptive analysis was performed, because of the difference in the outcome measures evaluated in the two studies.

*(1) Overall Scores on Scales Evaluating the Core Features of ASD*. Li and Liu [30] reported that the total CARS and ABC^1^ scores were lower when acupuncture was added to the combination therapy (both *P* < 0.01). Zeng et al. [48] reported that the TER calculated using the CARS was higher in the acupuncture group (*P* = 0.008). 

*(2) Social Interaction Skills.* One study [48] reported that social interaction skills were not significantly different between the two groups, when assessed by subscales of the PEP3 (*P* > 0.05). 

*(3) Communication Ability.* Zeng et al. [48] reported that communication abilities were not significantly different between the two groups, when assessed by subscales of the PEP3 (*P* > 0.05). 

*(4) Language Ability.* Both studies measured language ability, using either a subscale of the PEP3 [48] or the Gesell Developmental Schedules [30], and both showed a significant improvement in the acupuncture group compared to the control group (*P* < 0.05, *P* < 0.01; resp.). 

*(5) Cognitive Function.* One study [48] reported that cognitive function was higher in the acupuncture group compared with the control group, when assessed by subscales of the PEP3 (*P* = 0.0005).

### 3.4. Efficacy of Acupuncture as an Alternative Therapy

#### 3.4.1. Alternative Therapy to BEI

Four studies [28, 34, 44, 46] compared acupuncture with BEI. 

**Figure 5:**
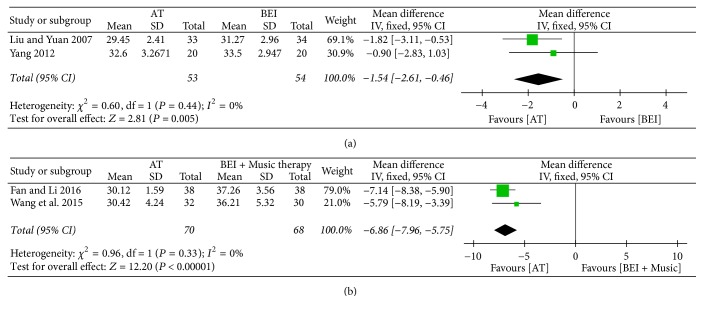
*Forest plots for the outcome “overall CARS score”. (a) Acupuncture versus BEI. (b) Acupuncture versus BEI and music therapy.* AT, acupuncture treatment; BEI, behavioral and educational interventions; CARS, Childhood Autism Rating Scale.

*(1) Overall Scores on Scales Evaluating the Core Features of ASD.* Two studies [34, 46] found greater improvement in the overall CARS score after treatment in the acupuncture group than in the BEI group (MD −1.54, 95% CI −2.61 to −0.46, *I*^2^ = 0%) (Figure 5).

The ABC^1^ [34], and the TER calculated with the CARS [46], also showed a significant improvement in the acupuncture group compared to the control group (both *P* < 0.05). 

*(2) Social Interaction Skills.* Yang [46] measured social interaction skills using a subscale of the CARS but found no significant difference between the two groups (*P* > 0.05). 

*(3) Communication Ability.* Yang [46] evaluated communication skills using subscales of the CARS. There were no significant differences in nonverbal communication abilities between the two groups (*P* > 0.05), but verbal communication abilities were significantly different in favour of the acupuncture group (*P* < 0.05). 

*(4) Language Ability.* Gao [28] reported that language ability was significantly improved in the acupuncture group compared with the BEI group (*P* < 0.05), when measured by a subscale of the PEP.

*(5) Cognitive Function.* In one study [28], there was no difference between interventions when cognitive function was evaluated by a subscale of the PEP (*P* > 0.05). 

#### 3.4.2. Alternative Therapy to BEI Combined with Music Therapy

Two studies [27, 39] compared acupuncture with a combination therapy consisting of both BEI and music therapy. Both studies showed improved total CARS scores in the acupuncture group compared with the control group, after treatment (MD −6.86, 95% CI −7.96 to −5.75, *I*^2^ = 0%) (Figure 5).

### 3.5. Acupuncture versus Sham Acupuncture

Two studies [41, 42] compared acupuncture plus BEI with sham acupuncture plus BEI. 

**Figure 6:**
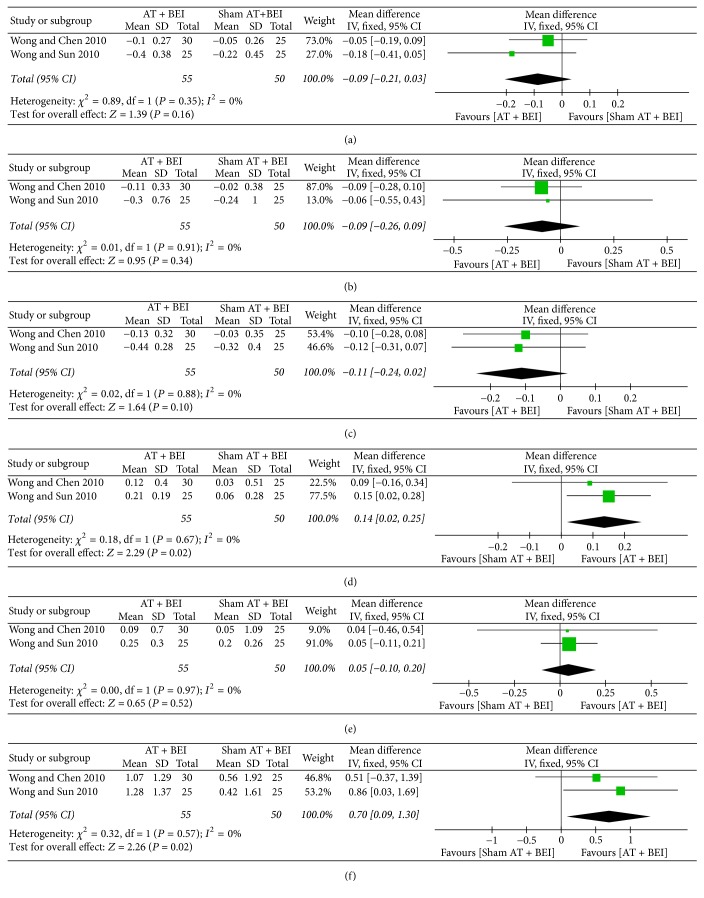
*Forest plots for comparison of acupuncture versus sham acupuncture. (a) Overall RFRLRS score. (b) Social interaction skill (RFRLRS). (c) Language ability (RFRLRS). (d) Language ability-comprehension age (RDLS). (e) Language ability-expression age (RDLS). (f) Cognitive function (WeeFIM).* AT, acupuncture treatment; BEI, behavioral and educational interventions; RDLS, Reynell Developmental Language Scales; RFRLRS, Ritvo-Freeman Real Life Rating Scale; WeeFIM, Functional Independence Measure for Children.

*(1) Overall Scores on Scales Evaluating the Core Features of ASD.* Both studies [41, 42] measured the total RFRLRS scores, which showed no significant differences between the two groups (MD −0.09, 95% CI −0.21 to 0.03, *I*^2^ = 0%) (Figure 6). 

*(2) Social Interaction Skills.* Social interaction skills were assessed using a subscale of the RFRLRS [41, 42] and the Pediatric Evaluation of Disability Inventory (PEDI) [41], but there were no significant differences between the groups (RFRLRS, MD −0.09, 95% CI −0.26 to 0.09, *I*^2^ = 0%; PEDI, *P* = 0.959) (Figure 6). 

*(3) Communication Ability.* One study [41] reported that there was no difference between the two groups when assessing communication ability using subscales of the ABC^2^ (*P* = 0.849). 

*(4) Stereotypy.* One study [41] evaluated stereotypy using subscales of the ABC^2^ and found no difference between the two groups (*P* = 0.629).

*(5) Language Ability.* There were no significant differences in language abilities between the two groups when evaluated using subscales of the RFRLRS [41, 42], RDLS [41, 42], and SPT [42] (RFRLRS, MD −0.11, 95% CI −0.24 to 0.02, *I*^2^ = 0%; RDLS-comprehension age, MD 0.14, 95% CI 0.02 to 0.25, *I*^2^ = 0%; RDLS-expression age, MD 0.05, 95% CI −0.10 to 0.20, *I*^2^ = 0%; SPT, *P* = 0.092) (Figure 6). 

*(6) Cognitive Function.* Both studies [41, 42] measured cognitive function using a subscale of the WeeFIM, which showed differences in favour of the acupuncture group (MD 0.70, 95% CI 0.09 to 1.30, *I*^2^ = 0%) (Figure 6). 

### 3.6. Other Effects of Acupuncture in ASD

In addition to the above-mentioned outcome measures, acupuncture has other potential effects on ASD. First, acupuncture might improve social adaptation. Li and Liu [30] found that participants who received acupuncture showed improved ability for social adaptation, as measured by the Gesell Developmental Schedules, compared to those who did not receive acupuncture (*P* < 0.01). Wang et al. [37] found that the social adaptive quotient and TER calculated were higher when acupuncture was added to BEI than for BEI alone (both *P* < 0.05).

Second, acupuncture might improve sleeping habits. In a study that evaluated the TER using the Children's Sleep Habits Questionnaire [26], the rate for the acupuncture plus BEI group was higher than that of the BEI-alone group (*P* = 0.001).

### 3.7. Safety

Six RCTs with 306 participants [35, 40–43, 49] provided data on adverse events associated with acupuncture (Table 3). Four trials [35, 42, 43, 49] reported that no adverse events occurred during the interventions. Wong and Chen [41] reported that some participants experienced minor superficial bleeding, crying, or irritability during acupuncture. Another study [40] reported one case of a worsening sleep pattern and one case of increased hyperactivity and ritualistic behavior.

## 4. Discussion

In the present study, we conducted a systematic review of 27 RCTs with 1736 participants, to evaluate the efficacy of acupuncture for children with ASD. The outcome assessment of our review was summarized in two aspects: improvement of overall symptoms of ASD (evaluated with the overall scores on scales such as CARS, ABC^1^, ABC^2^, ATEC, and RFRLRS, which are specific outcome measures for ASD) and improvement in each of the major symptom categories of ASD (including social interaction skills, communication ability, stereotypy, language ability, and cognitive function).

When acupuncture was added to BEI, our pooled results indicated that reductions in the overall scores on the CARS, ABC^1^, and ATEC, and TERs based on the overall scores on the CARS and ABC^1^, were significantly higher compared with the BEI-alone group. Acupuncture was not demonstrably effective in reducing the overall ABC^2^ and RFRLRS scores as an adjunctive therapy, but the results were unreliable because only a very small number of studies evaluated this. As a complementary therapy for BEI, acupuncture therapy provided inconsistent results in improving the individual symptoms of ASD. This may have been a result of evaluating each symptom using nonspecific scales for ASD, such as the SPT or WeeFIM. There were many studies that evaluated the total scores on ASD-specific outcome measures, but the scores on the subscales that evaluated each individual ASD symptom were rarely presented. Therefore, the number of studies evaluating each major symptom of ASD was insufficient for assessing statistical validity. The effect of acupuncture as a complementary therapy alongside pharmacotherapy, or as an adjunctive therapy to music therapy combined with BEI, could not be assessed because there were only one or two studies available.

When acupuncture was used as an alternative therapy for BEI, or for BEI plus music therapy, the total CARS scores of the acupuncture group showed greater improvement than those of the control groups. There was only one study evaluating the efficacy of acupuncture for each of the ASD symptoms, such as social interaction skills, communication ability, language ability, and cognitive function. Therefore, the results concerning the efficacy of acupuncture as a monotherapy for each symptom of ASD should be interpreted with caution.

Our review found that acupuncture had no significantly different effect on any outcome compared to sham acupuncture. In addition, while the results indicate that acupuncture might improve social adaptability and sleeping habits, these results cannot be confirmed because of the limited number of studies.

According to TCM's holistic approach, stimulating the acupoints related to various organs or viscera of the human body through meridians corrects the disharmony and dysregulation of organ systems. In TCM, the pathogenesis of ASD is considered to be related to disharmony of the five viscera, six bowels, and meridians, mainly dysregulation of the heart, liver, spleen, kidney, and brain [15]. Dysregulation and disharmony of the organs associated with ASD may have been corrected through acupuncture treatment, and this may have resulted in an improvement in overall scores on ASD-specific outcome measures, such as the CARS and ABC^1^. On a scientific basis, the pathogenesis of ASD is incompletely understood. Acupuncture can modulate neurotrophic factors [19] and neurotransmitter systems such as serotonin [53], GABA [17], and glutamate [16], which are considered strong candidates for roles in ASD [18], and this could lead to improvement in the symptoms of ASD. Further investigations should be conducted to clarify the specific mechanisms of acupuncture in treating ASD, and to examine which core symptoms of ASD can be improved by acupuncture.

We did not find strong evidence to show that acupuncture is associated with any serious adverse events. However, only six RCTs (22.22%) reported acupuncture safety data. Therefore, we could not draw a firm conclusion about the safety of acupuncture for ASD.

Two systematic reviews regarding acupuncture for ASD [20, 21] have been published during the last few years. Compared with the previous systematic reviews [20, 21], which included 10 and 11 articles, respectively, our review included many additional published RCTs. In other words, by including 27 RCTs and 1736 participants, this is the most comprehensive and recent review of acupuncture for patients with pediatric ASD. Unlike previous studies, which referred to the treatment used in the control group as “conventional treatment,” we analyzed the interventions used in the controls by classifying them as pharmacological interventions, BEI, and CAM. We could confirm that acupuncture as a complementary therapy improved the total CARS and ABC^1^ scores, which are tools for measuring the key symptoms of ASD, but could not specifically determine the symptoms that improved. The total CARS scores before and after treatment were reported in ten studies [27, 30, 32–36, 39, 45, 46], but there was only one study [46] that showed the scores on subscales evaluating each symptom. Meanwhile, we did not find much improvement in the low methodological quality of the included studies. Furthermore, owing to the heterogeneity of the acupuncture methods used in each study, we could not draw any other conclusions.

### 4.1. Limitations

This review has several limitations, and each should be considered seriously. First, we found that significant heterogeneity existed for several of the outcomes. This may have been caused by the heterogeneity of the recruited participants with ASD, as ASD comprises a range of symptoms and associated problems. Moreover, unlike other clinical trials, acupuncture clinical trials involve various factors that cause heterogeneity, such as selection of acupoints, frequency and duration of treatments, depths of insertion, and needle retention times. Although the treatments used in the control groups were classified in this review as pharmacological interventions, BEI, or CAM, the variety of BEI treatments may have caused heterogeneity. Second, the low methodological quality of some studies remains a challenge. Many studies showed performance bias, because acupuncture therapy is difficult to blind. The low methodological quality of some studies can lead to overestimation of the effects of acupuncture on ASD. Additionally, although we searched the literature regardless of language, most of the included studies were conducted in China (74.07%), which suggests a potential for location bias and limits the generality of this review. We intended to estimate publication bias with a funnel plot, but none of the outcome measures were used in more than 10 studies. Accordingly, we could not assess publication bias in this review.

### 4.2. Implications for Future Research

After the publication of the DSM-5 in 2013 [1], a single diagnosis of “ASD” has replaced previous subtypes. ASD is a highly heterogeneous disorder that involves various symptoms. Therefore, it is necessary to identify the subgroups of ASD children that can benefit from acupuncture. To resolve this in detail, subscales as well as total scores on the indicators evaluating ASD, such as the CARS, ABC^1^, and ABC^2^, should be measured and presented to distinguish the symptoms that might be improved by acupuncture. Only four of the studies included in this review [25, 36, 42, 43] used CARS scores as diagnostic criteria to specify the severity of the symptoms. In future studies, the severity of ASD should be specified in participants via appropriate inclusion criteria.

Since there were variations in the types of acupuncture used, and a lack of standardization in the selection of acupoints, future research must standardize and generalize acupuncture treatment for ASD. In the studies included in this review, GV20 and EX-HN3 were frequently used. The most frequent needle retention time was 30 minutes, the number of treatment sessions was 120, the frequency of treatment was 5 times/week, and the duration of treatment was 8 weeks. To accumulate evidence, we should select specific acupuncture treatment methods based on existing studies. Additionally, the included studies did not conduct follow-ups after the treatment ended. We suggest long-term clinical trials with longer follow-up periods that include serial measurements of outcomes to determine the effects of acupuncture over time.

To improve the methodological quality, a strict protocol should be planned prior to future trials. Moreover, we expect rigorous, well-designed clinical trials in full compliance with the Standards for Reporting Interventions in Clinical Trials of Acupuncture (STRICTA) recommendations [54]. In this review, only six studies reported adverse events related to acupuncture, and all events were mild. To draw clear conclusions about the safety of acupuncture for pediatric ASD, future studies should measure and report all adverse events using guidelines such as the CONSORT for Harms Data Recommendations [55]. Moreover, parents of children with ASD experience considerable levels of stress [56]; therefore, we suggest that the quality of life of parents of children with ASD should be considered as an additional important outcome measure in future clinical trials.

## 5. Conclusion

The results of this systematic review indicated that acupuncture may improve the overall symptoms of ASD. The reported adverse events associated with acupuncture were mild and acceptable. However, due to heterogeneities among the trials and the low methodological quality of some of the studies, additional rigorous, well-designed RCTs with larger sample sizes should be performed to confirm these results.

## Figures and Tables

**Table 1:** Summary of the studies included.

Study (reference)	Country	Sample size (A)/(B)	Mean age	Gender (M : F)	Diagnostic criteria	(A) Experimental intervention	(B) Control intervention	Acupuncture points	Outcomes
Allam et al. 2008 [25]	Egypt	10/10	5.5 ± 1.22 y	(A) 7 : 3 (B) 5 : 5	DSM-IV ADI-R CARS ≥ 30	MA + (B)	Language therapy	GV20, GV26, GV17, 3 temple needles, YNSA cerebrum and aphasia points	Arabic language test
Chen et al. 2014 [26]	China	30/30	5.06 ± 1.32 y	36 : 24	CCMD-3	MA + (B)	Cognitive behavior rehabilitation training	GV20, GV24, EX-HN3, HT7, SP6, HI3, head Anmian point	CSHQ, TER (CSHQ), PTQ
Fan and Li 2016 [27]	China	38/38	(A) 5.21 ± 1.56 y (B) 4.98 ± 2.05 y	(A) 22 : 16 (B) 20 : 18	NR	MA	Language therapy, guided education, cooperative exchanges, music therapy	Jin's three needle technique (Sishenzhen, Zhisanzhen, Naosanzhen, Niesanzhen, Shouzhizhen, Zuzhizhen, Shesanzhen, Xingshenzhen)	CARS, TER (language ability)
Gao 2015 [28]	Mongolia	50/50	(A) 5.7 ± 0.8 y (B) 5.4 ± 0.7 y	(A) 29 : 21 (B) 31 : 19	WHO	MA	Physical therapy, cognitive education, language education, behavior modification	Jin's three needle technique (Sishenzhen, Naosanzhen, Niesanzhen, Zuokeshangsanzhen, Dingshenzhen, Xingshenzhen, Shouzhizhen, Zuzhizhen, Shesanzhen)	PEP
He 2006 [29]	China	30/30	(A) 7.16 ± 4.26 y (B) 6.94 ± 3.86 y	(A) 24 : 6 (B) 25 : 5	DSM-IV ICD-10 CCMD-2R	EA + MA + (B)	Verbal communication training, social communication training, self-care training, cognitive training, motion training	EA (EX-HN1, EX-HN3, PC6, HT7, SP6, KI3), MA (LR3, AT3, TF4)	ABC^2^, CARS, ATEC
Li and Liu 2011 [30]	China	30/40	NR	(A) 27 : 3 (B) 33 : 7	DSM-IV CCMD	EA + (B)	Music therapy and structured teaching	Scalp points (Zhijiuzhen, emotional zone, heart and liver zone)	Clancy Autism Behavior Scale, CARS, ABC^1^, GDS
Li et al. 2011 [31]	China	19/19	42.75 ± 2.5 m	36 : 2	DSM-IV	MA + (B)	Behavioral educational intervention	Tongue points (Naozhongxue, Naoshuxue, Naoyuanxue, Bizhongxue)	PEP-revised
Li et al. 2015 [32]	China	53/53	5.1 ± 3.9 y	82 : 24	NR	MA + (B)	Intensive therapy, game therapy, sensory integration training	Jin's three needle technique (Shesanzhen, Zuzhizhen, Shouzhizhen, Xingshenzhen, Zhisanzhen, Naosanzhen, Niesanzhen, Dingshenzhen, Sishenzhen)	CARS, ABC^1^
Liao 2016 [33]	China	30/30	(A) 9.24 ± 1.64 y (B) 9.51 ± 1.50 y	(A) 19 : 11 (B) 18 : 12	NR	MA + (B)	ABA, language cognitive therapy, sensory integration training	Scalp temporal, frontal, suboccipital, occipital region	CARS, TER (CARS)
Liu and Yuan 2007 [34]	China	33/34	5.41 y	60 : 7	DSM-IV	MA	Sensory integration training	Jin's three needle technique (Sishenzhen, Niesanzhen, Zhisanzhen, Naosanzhen, Shesanzhen, Shouzhizhen, Zuzhizhen), Siguan	ABC^1^, CARS
J. G. Sun and X. R. Sun 2014 [35]	Hong Kong	42/42	(A) 70 m (B) 65 m	(A) 30 : 12 (B) 29 : 13	DSM-IV	EA + (B)	Behavior therapy, sensory integration training	Tongue points (Naozhongxue, Naoshuxue, Naoyuanxue, Bizhongxue)	CARS, TER (CARS)
Tang et al. 2013 [36]	China	20/18/18	(A1) 4.526 ± 1.623 y (A2) 4.436 ± 1.520 y (B) 3.954 ± 1.572 y	(A1) 14 : 6 (A2) 13 : 5 (B) 14 : 4	DSM-IV CCMD-2R CARS 36–41	(A1) doing (B) during retention of MA (A2) MA after (B)	Educational training, behavior modification, sensory integration training, language therapy	Scalp areas according to symptoms	CARS, TER (CARS)
Wang et al. 2006 [37]	China	30/30	NR	(A) 24 : 6 (B) 23 : 7	ICD-10 CCMD-3	EA + (B)	Behavior therapy	GV20, EX-HN1, GV24, GB13, EX-HN3, GV17, GB19, PC6, scalp speech areas 1, 2, 3	Social adaptive quotient, TER (social adaptive quotient)
Wang et al. 2007 [38]	China	30/30	(A) 5.14 ± 1.53 y (B) 5.46 ± 1.68 y	(A) 24 : 6 (B) 23 : 7	ICD-10 CCMD-3	EA + (B)	Sensory integration training, auditory integration training, language therapy	GV20, EX-HN1, GV24, GB13, EX-HN3, GV17, GB19, PC6, scalp speech areas 1, 2, 3	ABC^1^, TER (ABC^1^), PPVT
Wang et al. 2015 [39]	China	32/30	(A) 5.12 ± 1.42 y (B) 5.10 ± 3.4 y	(A) 18 : 14 (B) 15 : 15	NR	MA	Language therapy, guided education, ABA, music therapy	Jin's three needle technique (Sishenzhen, Zhisanzhen, Naosanzhen, Niesanzhen, Shouzhizhen, Zuzhizhen, Shesanzhen, Xingshenzhen)	CARS, TER (language ability)
Wong 2008^a^ [40]	Hong Kong	18/18	(A) 7.40 ± 2.215 y (B) 7.62 ± 2.367 y	(A) 17 : 1 (B) 17 : 1	DSM-IV ADI-R ADOS	EA + MA + (B)	Conventional educational program	EA (GV20, EX-HN3, HT7, SP6), MA (AT3)	ADOS, ABC^2^, ATEC, RFRLRS, WeeFIM, CGI, SPT
Wong and Chen 2010 [41]	Hong Kong	30/25	(A) 9.17 ± 4.12 y (B) 9.56 ± 4.22 y	(A) 25 : 5 (B) 22 : 3	DSM-IV ADI-R ADOS	EA, conventional educational program	Sham EA, conventional educational program	EX-HN1, EX-HN3, PC6, HT7, LR3, SP6, AT3, TF4	WeeFIM, PEDI, Leiter-R, CGI, ABC^2^, RFRLRS, RDLS, a standardized parental report
Wong and Sun 2010 [42]	Hong Kong	25/25	(A) 6.23 ± 1.8 y (B) 6 ± 1.99 y	(A) 21 : 4 (B) 23 : 2	DSM-IV ADI-R CARS ≥ 30	MA, conventional educational and behavior model	Sham MA, conventional educational and behavior model	Tongue points (Runze, Guanzhu, Tianmen, Diyou)	GMDS, RFRLRS, RDLS, SPT, WeeFIM
Wong et al. 2014 [43]	Hong Kong	16/11 (12/9)	(A) 10.167 ± 3.930 y (B) 8.750 ± 4.617 y	(A) 16 : 0 (B) 11 : 0	DSM-IV ADI-R CARS ≥ 30	MA + (B)	Conventional educational and behavior model	Tongue points (Runze, Guanzhu, Tianmen, Diyou)	ATEC, RDLS, SPT, WeeFIM, CGI, Cerebral FDG metabolism by PET
Wu 2015 [44]	China	40/40	(A) 6.5 ± 2.3 y (B) 6.2 ± 2.1 y	(A) 28 : 12 (B) 30 : 10	DSM-5	MA	Conventional intervention	Jin's three needle technique (NR)	Functional development scale
Xiong 2014 [45]	China	32/32	(A) 7.48 ± 3.26 y (B) 7.56 ± 3.12 y	(A) 18 : 14 (B) 17 : 15	CCMD-3	MA + (B)	Educational training, sensory training, behavior modification, language therapy	Scalp frontal, temporal, occipital, suboccipital, parietal regions	CARS
Yang 2012 [46]	China	20/20	(A) 5.6053 ± 2.2582 y (B) 4.2778 ± 1.8249 y	(A) 16 : 4 (B) 14 : 6	DSM-IV	MA	Behavior therapy, sensory integration training	Jin's three needle technique (Sishenzhen, Dingshenzhen, Zhisanzhen, Yansanzhen, Naosanzhen, Shesanzhen, Shousanzhen, Shouzhizhen, Zusanzhen, Zuzhizhen), CV12, CV4, CV6, GV14, BL20, GV4	CARS, TER (CARS), EEG, attention value
Zeng et al. 2014 [47]	China	30/25	(A) 3.51 ± 1.48 y (B) 3.49 ± 1.47 y	(A) 18 : 12 (B) 14 : 11	ICD-10	MA + (B)	ABA, TEACCH, PCI, sensory integration training, auditory integration training, language cognitive therapy	Jin's three needle technique (Sishenzhen, Naosanzhen, Niesanzhen, Zhisanzhen, Shesanzhen, Dingshenzhen, Shouzhizhen, Zuzhizhen)	TER (CARS), TER (PEP)
Zeng et al. 2016 [48]	China	60/25	(A) 3.62 ± 1.36 y (B) 3.60 ± 1.52 y	(A) 36 : 24 (B) 16 : 9	ICD-10	MA + (B)	ABA, TEACCH, PCI, sensory integration training, language cognitive therapy, music therapy	Jin's three needle technique (Naosanzhen, Niesanzhen, Zhisanzhen, Shesanzhen, Dingshenzhen, Shouzhizhen, Zuzhizhen)	TER (CARS), TER (PEP3)
Zhao et al. 2015 [49]	China	30/30	NR	NR	ICD-10	EA + (B)	Special education	Group 1: GV20, Sishenzhen, GV24, GB13 Group 2: ST8, GV23, Dingshenzhen, GV17, GB19; use group 1 or 2 alternately every other week	ABC^1^, TER (ABC^1^)
Zhao et al. 2015 [50]	China	35/30	(A) 3.5 y (B) 3.2 y	(A) 28 : 7 (B) 25 : 5	DSM-IV ICD-10	MA + (B)	Risperidone 0.5–2 mg/d	Jin's three needle technique (Dingshenzhen, Zhisanzhen, Sishenzhen, Niesanzhen), GV17, transport point mainly pericardium, heart, or liver meridian	TER (improvement of abnormal behavior)
Zhen and Jin 2016 [51]	China	68/68 (63/65)	(A) 4.2 y (B) 4.5 y	(A) 56 : 12 (B) 53 : 15	DSM-IV	MA + (B)	Speech training and structured teaching	Jin's three needle technique (Esanzhen, Niesanzhen, Zhensanzhen), EX-HN1	PEP-3

ABA, Applied Behavior Analysis; ABC^1^, Autism Behavior Checklist; ABC^2^, Aberrant Behavior Checklist; ADI-R, Autism Diagnostic Interview-Revised; ADOS, Autism Diagnostic Observation Scale; ATEC, Autism Treatment Evaluation Checklist; CARS, Childhood Autism Rating Scale; CCMD, Chinese Classification of Mental Disorder; CGI, Clinical Global Impression; CSHQ, Children's Sleep Habits Questionnaire; DSM, Diagnostic and Statistical Manual of Mental Disorders; EA, electroacupuncture; EEG, electroencephalography; FDG, fluorodeoxyglucose; GDS, Gesell Development Schedules; GMDS, Griffiths Mental Developmental Scale; ICD, International Classification of Diseases; MA, manual acupuncture; NR, not recorded; PCI, Play and Cultural Intervention; PEDI, Pediatric Evaluation of Disability Inventory; PEP, Psychoeducational Profile; PET, positron emission tomography; PPVT, Peabody Picture and Vocabulary Test; PTQ, Parent Temperament Questionnaire; RDLS, Reynell Developmental Language Scales; RFRLRS, Ritvo-Freeman Real Life Rating Scale; SPT, Symbolic Play Test; TEACCH, Treatment and Education of Autistic and Related Communication Handicapped Children; TER, Total Effective Rate; WeeFIM, Functional Independence Measure for Children; WHO, World Health Organization; YNSA, Yamamoto New Scalp Acupuncture; ^a^crossover trial. The rest are parallel studies.

**Table 2:** Details of acupuncture treatment methods.

Study (reference)	Depth of insertion	Deqi	Needle stimulation	Needle retention time	Number of treatment sessions	Frequency of treatment	Duration of treatment
Allam et al. 2008 [25]	0.5–1 cun^*∗*^	O	Rapid manual stimulation upon insertion and removal of the needles	20 min	NR	Twice per week	9 months
Chen et al. 2014 [26]	NR^+^	NR	Han's acupoint nerve stimulator	30 min	NR	5 times per week	2 months
Fan and Li 2016 [27]	NR	NR	Twirling every 30 min	Head: 1 h, others: 30 min	20	5 times per week	NR
Gao 2015 [28]	NR	NR	Twirling every 5 min	45 min	NR	6 times per week	20 weeks
He 2006 [29]	NR	NR	Electrical stimulation (10 V, wave of rarefaction)	NR	24	3 times per week	8 weeks
Li and Liu 2011 [30]	0.5–1 cun	NR	Electrical stimulation (1.25 Hz, wave of condensation and rarefaction, 15 min), twirling	2–4 h	60	6 times per week	NR
Li et al. 2011 [31]	NR	NR	Twirling, lifting, thrusting	No retention	NR	1–5 times per week	6 weeks
Li et al. 2015 [32]	NR	NR	Twirling every 10 min	30 min	NR	6 times per week	3 months
Liao 2016 [33]	10–15 mm	NR	Twirling every 30 min	3-4 h	NR	NR	NR
Liu and Yuan 2007 [34]	Head: 0.5–0.8 cun, tongue, limbs: 0.2–0.8 cun	NR	Twirling every 10 min	30 min	72	6 times per week	12 weeks
J. G. Sun and X. R. Sun 2014 [35]	NR	NR	Electrical and thermal stimulation	20 min	NR	3 times per day	2 months
Tang et al. 2013 [36]	10–15 mm	NR	Twirling	6–8 h	NR	6 times per week	25 weeks
Wang et al. 2006 [37]	GV20, EX-HN1: 0.5–0.8 cun	O	Electrical stimulation (1.25 Hz, wave of condensation and rarefaction)	50 min	NR	5 times per week	4 months
Wang et al. 2007 [38]	GV20: 15–20 mm, EX-HN1: 1.5–20 mm	O	Electrical stimulation (75 Hz)	50 min	NR	5 times per week	4 months
Wang et al. 2015 [39]	NR	NR	Twirling	Head: 1 h, limbs: 30 min	20	5 times per week	NR
Wong 2008^a^ [40]	GV20, EX-HN3, HT7, SP6: 13–25 mm, AT3: 5–10 mm	O	Electrical stimulation (25–75 Hz, continuous wave)	30 min	24	3 times per week	8 weeks
Wong and Chen 2010 [41]	NR	NR	Electrical stimulation	30 min	12	3 times per week	4 weeks
Wong and Sun 2010 [42]	Runze: 1 cm, Guanzhu: 0.3 cm, Tianmen: 0.5–1 cm, Diyou: 0.5 cm	NR	Quick and accurate insertion into acupoints	<15 s	40	5 times per week	8 weeks
Wong et al. 2014 [43]	Runze: 1 cm, Guanzhu: 0.3 cm, Tianmen: 0.5–1 cm, Diyou: 0.5 cm	NR	Quick and accurate insertion into acupoints	<15 s	40	5 times per week	8 weeks
Wu 2015 [44]	NR	NR	NR	NR	120	6 times per week	NR
Xiong 2014 [45]	10–15 mm	NR	Twirling every 1 h	6–8 h	NR	NR	NR
Yang 2012 [46]	Trunk: 0.1–0.3 cun, head: 0.5–0.8 cun	NR	Twirling every 20 min	60 min, trunk: no retention	120	5 times per week	NR
Zeng et al. 2014 [47]	NR	NR	Twirling every 15 min	1 h	120	5 times per week	6 months
Zeng et al. 2016 [48]	Head: 15–20 mm, tongue, limbs: 5–15 mm	O	Twirling every 15 min	1 h	120	5 times per week	6 months
Zhao et al. 2015 [49]	25 mm	NR	Electrical stimulation (1–100 Hz, continuous wave, ≤50 mA, 30 min)	2 h	NR	6 times per week	3 months
Zhao et al. 2015 [50]	1–1.5 cun	NR	Twirling 200 times per min for 2-3 min	Scalp: 1 h, others: no retention	NR	6 times per week	3 months
Zhen and Jin 2016 [51]	NR	NR	Lifting and thrusting	30 min	NR	5 times per week	3 months

^*∗*^1 cun  = 3.0303030 cm; ^+^NR: not recorded. ^a^Crossover trial. The rest are parallel studies.

**Table 3:** Adverse events in included studies.

Study (reference)	Sample size (E/C)	Adverse events
J. G. Sun and X. R. Sun 2014 [35]	42/42	None
Wong 2008 [40]	18/18	E: 1 (worsening of sleep pattern; sleeping late at night), 1 (worsening of hyperactivity and ritualistic behavior) Parents thought that the negative changes were minor and did not affect the participant's functioning
Wong and Chen 2010 [41]	30/25	E: minor superficial bleeding or crying, and irritability during acupuncture was experienced by some
Wong and Sun 2010 [42]	25/25	None. Initial crying in fear and possible minor pain occurred in the first few sessions, but patients adapted easily and tolerated the procedure well
Wong et al. 2014 [43]	12/9	None. Initial crying in fear and possible minor pain occurred in first few sessions, but patients adapted easily and tolerated the procedure well
Zhao et al. 2015 [49]	30/30	None

C, control group; E, experimental group.

## Supplementary Materials

Supplementary Materials Supplementary Material includes the search terms for each database described in Methods. Supplement 1: search strategy used in all databases.
