# Corrigendum to “The Efficacy and Safety of Acupuncture for the Treatment of Children with Autism Spectrum Disorder: A Systematic Review and Meta-Analysis”

**DOI:** 10.1155/2023/9840285

**Published:** 2023-02-04

**Authors:** Boram Lee, Jihong Lee, Jin-Hong Cheon, Hyun-Kyung Sung, Seung-Hun Cho, Gyu Tae Chang

**Affiliations:** ^1^Department of Clinical Korean Medicine, Graduate School, Kyung Hee University, Kyung Hee Dae-ro 26, Dongdaemun-gu, Seoul 02447, Republic of Korea; ^2^Department of Korean Pediatrics, Hospital of Korean Medicine, Pusan National University, Geumo-ro 20, Mulgeum-eup, Yangsan-si, Gyeongsangnam-do 50612, Republic of Korea; ^3^Department of Pediatrics, College of Korean Medicine, Hospital of Semyung University, St. Sangbang 4-63, Chungju City, Chungcheongbuk-do 27429, Republic of Korea; ^4^Department of Neuropsychiatry, College of Korean Medicine, Kyung Hee University, Kyung Hee Dae-ro 26, Dongdaemun-gu, Seoul 02447, Republic of Korea

In the article titled “The Efficacy and Safety of Acupuncture for the Treatment of Children with Autism Spectrum Disorder: A Systematic Review and Meta-Analysis” [1], the authors wish to correct six errors in the countries listed in Table 1. The revised Table 1 is as follows:

## Figures and Tables

**Table 1:** Summary of the studies included.

Study (reference)	Country	Sample size (A)/(B)	Mean age	Gender (M : F)	Diagnostic criteria	(*A*) Experimental intervention	(*B*) Control intervention	Acupuncture points	Outcomes
Allam [25]	Egypt	10/10	5.5 ± 1.22 y	(*A*) 7 : 3 (*B*) 5 : 5	DSM-IV ADI-R CARS ≥30	MA + (B)	Language therapy	GV20, GV26, GV17, 3 temple needles, YNSA cerebrum, and aphasia points	Arabic language test
Chen [26]	China	30/30	5.06 ± 1.32 y	36 : 24	CCMD-3	MA + (B)	Cognitive behavior rehabilitation training	GV20, GV24, EX-HN3, HT7, SP6, HI3, head Anmian point	CSHQ, TER (CSHQ), PTQ
Fan [27]	China	38/38	(*A*) 5.21 ± 1.56 y (*B*) 4.98 ± 2.05 y	(*A*) 22 : 16 (*B*) 20 : 18	NR	MA	Language therapy, guided education, cooperative exchanges, music therapy	Jin's three needles (Sishenzhen, Zhisanzhen, Naosanzhen, Niesanzhen, Shouzhizhen, Zuzhizhen, Shesanzhen, Xingshenzhen)	CARS, TER (language ability)
Gao [28]	China	50/50	(*A*) 5.7 ± 0.8 y (*B*) 5.4 ± 0.7 y	(*A*) 29 : 21 (*B*) 31 : 19	WHO	MA	Physical therapy, cognitive education, language education, behavior modification	Jin's three needles (Sishenzhen, Naosanzhen, Niesanzhen, Zuonieshangsanzhen, Dingshenzhen, Xingshenzhen, Shouzhizhen, Zuzhizhen, Shesanzhen)	PEP
He [29]	China	30/30	(*A*) 7.16 ± 4.26 y (*B*) 6.94 ± 3.86 y	(*A*) 24 : 6 (*B*) 25 : 5	DSM-IV ICD-10 CCMD-2R	EA + MA + (B)	Verbal communication training, social communication training, self-care training, cognitive training, motion training	EA (EX-HN1, EX-HN3, PC6, HT7, SP6, KI3), MA (LR3, AT3, TF4)	ABC^2^, CARS, ATEC
Li [30]	China	30/40	NR	(*A*) 27 : 3 (*B*) 33 : 7	DSM-IV CCMD	EA + (B)	Music therapy and structured teaching	Scalp points (Zhijiuzhen, emotional zone, heart, and liver zone)	Clancy autism behavior scale, CARS, ABC^1^, GDS
Li [31]	China	19/19	42.75 ± 2.5 m	36 : 2	DSM-IV	MA + (B)	Behavioral educational intervention	Tongue points (Naozhongxue, Naoshuxue, Naoyuanxue, Bizhongxue)	PEP-revised
Li [32]	China	53/53	5.1 ± 3.9 y	82 : 24	NR	MA + (B)	Intensive therapy, game therapy, sensory integration training	Jin's three needle (Shesanzhen, Zuzhizhen, Shouzhizhen, Xingshenzhen, Zhisanzhen, Naosanzhen, Niesanzhen, Dingshenzhen, Sishenzhen)	CARS, ABC^1^
Liao [33]	China	30/30	(*A*) 9.24 ± 1.64 y (*B*) 9.51 ± 1.50 y	(*A*) 19 : 11 (*B*) 18 : 12	NR	MA + (B)	ABA, language cognitive therapy, sensory integration training	Scalp temporal, frontal, suboccipital, occipital region	CARS, TER (CARS)
Liu [34]	China	33/34	5.41 y	60 : 7	DSM-IV	MA	Sensory integration training	Jin's three needles (Sishenzhen, Niesanzhen, Zhisanzhen, Naosanzhen, Shesanzhen, Shouzhizhen, Zuzhizhen, Siguanxue), Siguan	ABC^1^, CARS
Sun [35]	China	42/42	(*A*) 70 m (*B*) 65 m	(*A*) 30 : 12 (*B*) 29 : 13	DSM-IV	EA + (B)	Behavior therapy, sensory integration training	Tongue points (Naozhongxue, Naoshuxue, Naoyuanxue, Bizhongxue)	CARS, TER (CARS)
Tang [36]	China	20/18/18	(*A*1) 4.526 ± 1.623 y (*A*2) 4.436 ± 1.520 y (*B*) 3.954 ± 1.572 y	(*A*1) 14 : 6 (*A*2) 13 : 5 (*B*) 14 : 4	DSM-IV CCMD-2R CARS 36-41	(A1) doing (B) during retention of MA (A2) MA after (B)	Educational training, behavior modification, sensory integration training, language therapy	Scalp areas according to symptoms	CARS, TER (CARS)
Wang [37]	China	30/30	NR	(*A*) 24 : 6 (*B*) 23 : 7	ICD-10 CCMD-3	EA + (B)	Behavior therapy	GV20, EX-HN1, GV24, GB13, EX-HN3, GV17, GB19, PC6, scalp speech area 1, 2, 3	Social adaptive quotient, TER (social adaptive quotient)
Wang [38]	China	30/30	(*A*) 5.14 ± 1.53 y (*B*) 5.46 ± 1.68 y	(*A*) 24 : 6 (*B*) 23 : 7	ICD-10 CCMD-3	EA + (B)	Sensory integration training, auditory integration training, language therapy	GV20, EX-HN1, GV24, GB13, EX-HN3, GV17, GB19, PC6, scalp speech area 1, 2, 3	ABC^1^, TER (ABC^1^), PPVT
Wang [39]	China	32/30	(*A*) 5.12 ± 1.42 y (*B*) 5.10 ± 3.4 y	(*A*) 18 : 14 (*B*) 15 : 15	NR	MA	Language therapy, guided education, ABA, music therapy	Jin's three needles (Sishenzhen, Zhisanzhen, Naosanzhen, Niesanzhen, Shouzhizhen, Zuzhizhen, Shesanzhen, Xingshenzhen)	CARS, TER (language ability)
Wong [40]	China	18/18	(*A*) 7.40 ± 2.215 y (*B*) 7.62 ± 2.367 y	(*A*) 17 : 1 (*B*) 17 : 1	DSM-IV ADI-R ADOS	EA + MA + (B)	Conventional educational program	EA (GV20, EX-HN3, HT7, SP6), MA (AT3)	ADOS, ABC^2^, ATEC, RFRLRS, WeeFIM, CGI, SPT
Wong [41]	China	30/25	(*A*) 9.17 ± 4.12 y (*B*) 9.56 ± 4.22 y	(*A*) 25 : 5 (*B*) 22 : 3	DSM-IV ADI-R ADOS	EA, conventional educational program	Sham EA, conventional educational program	EX-HN1, EX-HN3, PC6, HT7, LR3, SP6, AT3, TF4	WeeFIM, PEDI, Leiter-R, CGI, ABC^2^, RFRLRS, RDLS, A standardized parental report
Wong [42]	China	25/25	(*A*) 6.23 ± 1.8 y (*B*) 6 ± 1.99 y	(*A*) 21 : 4 (*B*) 23 : 2	DSM-IV ADI-R CARS ≥30	MA, conventional educational and behavior model	Sham MA, conventional educational and behavior model	Tongue points (Runze, Guangzhou, Tianmen, Diyou)	GMDS, RFRLRS, RDLS, SPT, WeeFIM
Wong [43]	China	16/11 (12/9)	(*A*) 10.167 ± 3.930 y (*B*) 8.750 ± 4.617 y	(*A*) 16 : 0 (*B*) 11 : 0	DSM-IV ADI-R CARS ≥30	MA + (B)	Conventional educational and behavior model	Tongue points (Runze, Guangzhou, Tianmen, Diyou)	ATEC, RDLS, SPT, WeeFIM, CGI, Cerebral FDG metabolism by PET
Wu [44]	China	40/40	(*A*) 6.5 ± 2.3 y (*B*) 6.2 ± 2.1 y	(*A*) 28 : 12 (*B*) 30 : 10	DSM-5	MA	Conventional intervention	Jin's three needles (NR)	Functional development scale
Xiong [45]	China	32/32	(*A*) 7.48 ± 3.26 y (*B*) 7.56 ± 3.12 y	(*A*) 18 : 14 (*B*) 17 : 15	CCMD-3	MA + (B)	Educational training, sensory training, behavior modification, language therapy	Scalp frontal, temporal, occipital, suboccipital, and parietal regions	CARS
Yang [46]	China	20/20	(*A*) 5.6053 ± 2.2582 y (*B*) 4.2778 ± 1.8249 y	(*A*) 16 : 4 (*B*) 14 : 6	DSM-IV	MA	Behavior therapy, sensory integration training	Jin's three needles (Sishenzhen, Dingshenzhen, Zhisanzhen, Yansanzhen, Naosanzhen, Shesanzhen, Shousanzhen, Shouzhizhen, Zusanzhen, Zuzhizhen), CV12, CV4, CV6, GV14, BL20, GV4	CARS, TER (CARS), EEG, attention value
Zeng [47]	China	30/25	(*A*) 3.51 ± 1.48 y (*B*) 3.49 ± 1.47 y	(*A*) 18 : 12 (*B*) 14 : 11	ICD-10	MA + (B)	ABA, TEACCH, PCI, sensory integration training, auditory integration training, language cognitive therapy	Jin's three needles (Sishenzhen, Naosanzhen, Niesanzhen, Zhisanzhen, Shesanzhen, Dingshenzhen, Shouzhizhen, Zuzhizhen)	TER (CARS), TER (PEP)
Zeng [48]	China	60/25	(*A*) 3.62 ± 1.36 y (*B*) 3.60 ± 1.52 y	(*A*) 36 : 24 (*B*) 16 : 9	ICD-10	MA + (B)	ABA, TEACCH, PCI, sensory integration training, language cognitive therapy, music therapy	Jin's three needles (Naosanzhen, Niesanzhen, Zhisanzhen, Shesanzhen, Dingshenzhen, Shouzhizhen, Zuzhizhen)	TER (CARS), TER (PEP3)
Zhao [49]	China	30/30	NR	NR	ICD-10	EA + (B)	Special education	Group 1: GV20, Sishenzhen, GV24, GB13 Group 2: ST8, GV23, Dingshenzhen, GV17, GB19; use group 1 or 2 alternately every other week	ABC^1^, TER (ABC^1^)
Zhao [50]	China	35/30	(*A*) 3.5 y (*B*) 3.2 y	(*A*) 28 : 7 (*B*) 25 : 5	DSM-IV ICD-10	MA + (B)	Risperidone 0.5–2 mg/d	Jin's three needles (Dingshenzhen, Zhisanzhen, Sishenzhen, Niesanzhen), GV17, transport point mainly pericardium, heart, or liver meridian	TER (Improvement of abnormal behavior)
Zhen [51]	China	68/68 (63/65)	(*A*) 4.2 y (*B*) 4.5 y	(*A*) 56 : 12 (*B*) 53 : 15	DSM-IV	MA + (B)	Speech training and structured teaching	Jin's three needles (Esanzhen, Niesanzhen, Zhensanzhen), EX-HN1	PEP-3

ABA, applied behavior analysis; ABC^1^, autism behavior checklist; ABC^2^, aberrant behavior checklist; ADI-R, autism diagnostic interview-revised; ADOS, autism diagnostic observation scale; ATEC, autism treatment evaluation checklist; CARS, childhood autism rating scale; CCMD, Chinese classification of mental disorder; CGI, clinical global impression; CSHQ, children's sleep habits questionnaire; DSM, diagnostic and statistical manual of mental disorders; EA, electroacupuncture; EEG, electroencephalography; FDG, fluorodeoxyglucose; GDS, gesell developmental schedules; GMDS, griffiths mental developmental scale; ICD, international classification of diseases; MA, manual acupuncture; NR, not recorded; PCI, play and cultural intervention; PEDI, pediatric evaluation of disability inventory; PEP, psychoeducational profile; PET, positron emission tomography; PPVT, peabody picture and vocabulary test; PTQ, parent temperament questionnaire; RDLS, reynell developmental language scales; RFRLRS, ritvo-freeman real life rating scale; SPT, symbolic play test; TEACCH, treatment and education of autistic and related communication handicapped children; TER, total effective rate; WeeFIM, functional independence measure for children; WHO, World Health Organization; YNSA, Yamamoto new scalp acupuncture. ^a^Crossover trial. The rest are parallel studies.
