# Supplementary material for: The Efficacy and Safety of Acupuncture for the Treatment of Children with Autism Spectrum Disorder: A Systematic Review and Meta-Analysis
Source: Evid Based Complement Alternat Med. 2018 Jan 11;2018:1057539. doi: 10.1155/2018/1057539 (PMC5820575; doi:10.1155/2018/1057539)
Supplement: Supplementary Materials — Supplementary Material includes the search terms for each database described in Methods. Supplement 1: search strategy used in all databases. [file 1057539.f1.docx]

**Supplementary Material**

**Supplement 1. Search Strategy Used in All Databases**

*Cochrane library, MEDLINE, AMED, CINAHL, KoreaMed*

(autis* OR pervasive developmental disorder* OR childhood disintegrative disorder OR asperger* OR autism spectrum disorder OR child development disorders, pervasive) AND (acup* OR needl* OR trigger point OR body acupuncture OR scalp acupuncture OR tongue acupuncture OR auricular acupuncture OR pharmacopuncture OR bee OR acupuncture OR acupuncture therapy OR acupuncture points OR acupuncture, ear OR electroacupuncture)

*EMBASE*

(‘autism’/exp OR 'autism' OR ‘asperger syndrome’/exp OR 'asperger syndrome' OR ‘autism spectrum disorder’ OR ‘child development disorders, pervasive’) AND (‘acupuncture’/exp OR ‘acupuncture’ OR ‘acupuncture needle’/exp OR 'acupuncture needle' OR ‘trigger point’/exp OR 'trigger point' OR 'bee venom' OR ‘bee venom’/exp OR ‘apitherapy’ OR ‘apitherapy’/exp OR ‘acupuncture therapy’ OR ‘acupuncture points’ OR ‘acupuncture, ear’ OR ‘electroacupuncture’)

*PsycARTICLES*

("autis*" OR "pervasive developmental disorder*" OR "childhood disintegrative disorder" OR "asperger*" OR "autism spectrum disorder" OR "child development disorders, pervasive") AND ("acup*" OR "needl*" OR "trigger point" OR "body acupuncture" OR "scalp acupuncture" OR "tongue acupuncture" OR "auricular acupuncture" OR "pharmacopuncture" OR "bee" OR "acupuncture" OR "acupuncture therapy" OR "acupuncture points" OR "acupuncture, ear" OR "electroacupuncture")

*CNKI*

(SU=’自闭症谱系障碍’+’全身性发育迟缓’+’蔓延性发育障碍’+’自闭’+’自闭性’+’瓦解性障碍’+’蜕变性障碍’+’肌腔隙障碍’+’孤独症’+’孤独性障碍’+‘自闭症’+’广泛性发育障碍’+’崩解症’+’Heller’+’瓦解性精神障碍’+’婴儿痴呆’+’衰退性精神病’+’自我中心主义‘+’泛自闭症障碍‘+’自闭症类群‘+’泛自闭症候群‘+**’**孤独症谱系障碍**’**+“autism”+“autistic”+“asperger”+“disintegrative”+“pervasive”+“autism spectrum disorder”+“child development disorders, pervasive”) and (SU=’针’+’针刺’+’针法‘+’刺法‘+’温针‘+’温针疗法‘+’火针‘+’刀针‘+’埋针‘+’埋针法‘+’头针’+’舌针’+’耳针’+’电针’+’电针刺‘+’电针疗法‘+’蜂毒‘+’蜂疗‘+’水针’+’穴位注射‘+’穴位注射疗‘+’针药治疗‘+’穴位’+’针压’+“acupoint”+“needling”+“needle”+“trigger point”+“body acupuncture”+“scalp acupuncture”+“tongue acupuncture”+“auricular acupuncture”+ “bee”+ “pharmacopuncture”+“acupuncture”+“acupuncture therapy”+“acupuncture points”+“acupuncture, ear” +“electroacupuncture”)

*Wanfang Data*

(“自闭症谱系障碍”+“全身性发育迟缓”+“蔓延性发育障碍”+“自闭”+“自闭性”+“瓦解性障碍”+“蜕变性障碍”+“肌腔隙障碍”+“孤独症”+“孤独性障碍”+“自闭症”+“广泛性发育障碍”+“崩解症”+“瓦解性精神障碍”+“婴儿痴呆”+“衰退性精神病”+“自我中心主义”+“泛自闭症障碍”+“自闭症类群”+“泛自闭症候群”+“孤独症谱系障碍”) * (“针”+“针刺”+“针法”+“刺法”+“温针”+“温针疗法”+“火针”+“刀针”+“埋针”+“埋针法”+“穴位”+“针压”+“头针”+“舌针”+“耳针”+“电针”+“电针刺”+“电针疗法”+“蜂毒”+“蜂疗”+“水针”+“穴位注射”+“穴位注射疗”+“针药治疗”)

*JAIRO*

(自閉 | 広汎性発達障害 | 小児期崩壊性障害 | アスペルガ | autis* | pervasive developmental disorder | childhood disintegrative disorder | asperger* | autism spectrum disorder | child development disorders, pervasive) (鍼 | ツボ | 指圧 | 頭鍼 | 舌鍼 | 耳鍼 | 電針 | 穴位注射 | acup* | needl* | trigger point | body acupuncture | scalp acupuncture | tongue acupuncture | auricular acupuncture | pharmacopuncture | bee | acupuncture | acupuncure therapy | acupuncture points | acupuncture, ear | electroacupuncture)

*CiNii*

(自閉 OR 広汎性発達障害 OR 小児期崩壊性障害 OR アスペルガ OR autis* OR pervasive developmental disorder OR childhood disintegrative disorder OR asperger* OR autism spectrum disorder OR child development disorders, pervasive) AND (鍼 OR ツボ OR 指圧 OR 頭鍼 OR 舌鍼 OR 耳鍼 OR 電針 OR 穴位注射 OR acup* OR needl* OR trigger point OR body acupuncture OR scalp acupuncture OR tongue acupuncture OR auricular acupuncture OR pharmacopuncture OR bee OR acupuncture OR acupuncture therapy OR acupuncture points OR acupuncture, ear OR electroacupuncture)

*OASIS*

(자폐 OR 전반적 발달장애 OR 소아기 붕괴성 장애 OR 아스퍼거 OR autism OR autistic OR asperger OR pervasive OR disintegrative OR autism spectrum disorder OR child development disorders, pervasive) AND (침 OR 혈위 OR 지압 OR 체침 OR 두침 OR 설침 OR 이침 OR 전침 OR 온침 OR 화침 OR 도침 OR 매침 OR 약침 OR 봉침 OR 봉독 OR pharmacopuncture OR bee OR body acupuncture OR scalp acupuncture OR tongue acupuncture OR auricular acupuncture OR acupoint OR needling OR needle OR trigger point OR acupuncture OR acupuncture therapy OR acupuncture points OR acupuncture, ear OR electroacupuncture)

*KTKP*

(자폐 | 전반적 발달장애 | 소아기 붕괴성 장애 | 아스퍼거 | autism | autistic | asperger | pervasive | disintegrative | autism spectrum disorder | child development disorders, pervasive) & (침 | 혈위 | 지압 | 두침 | 설침 | 이침 | 전침 | 약침 | 봉침 | 봉독 | 온침 | 화침 | 도침 | 매침 | scalp acupuncture | tongue acupuncture | auricular acupuncture | acupoint | needling | needle | trigger point | acupuncture | acupuncture therapy | acupuncture Points | acupuncture, ear | electroacupuncture | pharmacopuncture | bee)
